# Breaking law of overburden rock and key mining technology for narrow coal pillar working face in isolated island

**DOI:** 10.1038/s41598-024-63814-1

**Published:** 2024-06-06

**Authors:** Du Feng, Li Zhenhua, Li Songtao, Li Xiaolei, Li Guodong, Fan Xuan, Ren Hao, Cao Zhengzheng

**Affiliations:** 1 School of Energy Science and Engineering, Henan Polytechnic University, Jiaozuo, 454000 Henan China; 2 Collaborative Innovation Center of Coal Work Safety and Clean High Efficiency Utilization, Jiaozuo, 454000 Henan China; 3 Henan Mine Water Disaster Prevention and Control and Water Resources Utilization Engineering Technology Research Center, Henan Polytechnic University, Jiaozuo, 454000 Henan China; 4 Zhaogu No.2 Mine, Henan Energy Group Coking Coal Company, Jiaozuo, 454000 Henan China; 5 International Joint Research Laboratory of Henan Province for Underground Space Development and Disaster Prevention, Henan Polytechnic University, Jiaozuo, 454000 Henan China

**Keywords:** Segmental coal pillar, Overburden structure, Stress arch, Bearing structure, Mechanical modelling, Coal, Civil engineering

## Abstract

When conducting retreat mining in segmented coal pillars, the dynamic evolution of stress and overlying strata structure is more complex than conventional working faces due to the influence of adjacent working faces. Understanding and mastering the dynamic evolution patterns of overlying strata structure after retreat mining in segmented coal pillar working faces is essential for guiding the safe recovery of coal pillar resources under similar conditions. Through statistical analysis of the types of residual coal and the mining techniques, the current research status of residual coal mining system in China has been summarized. Based on the safety recovery technology system for multi-type residual coal pillar resources at Zhaogu No.2 Mine, this paper focuses on narrow coal pillar working faces in sections with fully mined-out areas on both sides. By using research methods such as on-site measurement, theoretical analysis, numerical simulation, and engineering experiments, starting from the stress state analysis and evolution law of coal seam mining, the dynamic evolution law of the overlying rock structure of sectional coal pillars has been mastered. On this basis, a stress arch mechanical model of the mining area is constructed, and the working resistance of the support is calculated and determined, ensuring the safe recovery of the working face. The research results show that before the backfilling of the sectional coal pillar working face, the working face is affected by the overlapping mining of the goaf on both sides, presenting a “bimodal” stress distribution pattern, with a stress concentration coefficient between 1.78 and 3.2. After the extraction of segmented coal pillars, stress arches consisting of high-stress zones form along both the strike and dip of the working face. The structural support provided by stress arches undergoes a dynamic evolution process of “formation-development-elevation-stabilization” as the working face advances. Following the instability and rupture of the lower basic roof hinge structure, the stress-bearing structure shifts to the higher basic roof, continuing to provide support for the surrounding rock stress in the mining space of the working face. A stress arch mechanical model for the dip and strike of the mining area is constructed , and the shape characteristics of the overlying rock stress arch in the coal pillar working face is mastered. Based on the stress distribution law and stress arch evolution characteristics of the surrounding rock of the coal pillar working face, the maximum working resistance of the support in the working face is theoretically calculated to be 9153.48kN. Compared with the measured mine pressure data, the selected support effectively ensures the safety production of the working face.

## Introduction

As the cornerstone of Chinese energy system and a solid support for national energy security, coal plays a crucial role in energy supply that cannot be ignored^1^. In actual mining operations, to ensure safe and efficient extraction of working faces, coal mines adopt the method of leaving segmented coal pillars to isolate mined-out areas and maintain the stability of the extraction drifts^2,3^. Simultaneously, this practice also results in the abandonment of a large quantity of high-quality coal resources, posing a serious threat to the sustainability of coal mining^4,5^. Therefore, the safe and efficient extraction of residual coal pillar resources, which are abundant in reserves and play a promoting role in the sustainable development of mines, remains a significant engineering challenge that urgently needs to be addressed.

Before the sectional coal pillar is mined back, under the influence of superimposed mining on both sides of the working face, the stress evolution characteristics of the rock body in the quarry and the form of rock breakage show diversity^6,7^. Therefore, it is of great significance to clarify the transport law of the overlying rock of the coal pillar in the section to guide how to control the mineral pressure of the coal pillar and the safe mining of the working face. At present, scholars at home and abroad have carried out a large number of researches on the theoretical study models for the movement of the overburden rock structure of the isolated working face, which mainly include the beam-rock, rock-slab, and stress arch structural models^8–13^.

Dou et al^14^, Zhu et al^15^, He^16^, and Wang^17^ classified and studied various forms of overlying space structures formed after the extraction of isolated working faces, proposing structures such as F-type and T-type, which are of guiding significance for studying the complex spatial structures of isolated working faces. Cao et al^18^, Cheng et al^19^, Hou et al^20^ studied the fracture structures of isolated working faces on a horizontal level, proposing “O,” “OX,” and “C” type overlying fracture structures, providing theoretical foundations and technical means for controlling dynamic disasters in mines. Jiang et al^21^ proposed the concept of the overlying space structure of isolated working faces based on the different boundaries of the isolated working face gob. According to the different boundaries of the isolated working face gob, the overlying space structure is classified as follows: ① Four-sided gob with supported “θ” type spatial structure; ② One-sided gob with unsupported “O” type spatial structure; ③ Two-sided gob with “S” type spatial structure; ④ Three-sided gob with “C” type spatial structure. Jiang et al^22^, Feng et al^23^ introduced the concepts of fully suspended roof structure, semi-suspended roof structure, and fully mobile structure for the roof structure of isolated mining faces. They analyzed the multi-layer spatial movement of the overlying rock mass and its relationship with the stress field induced by mining. Chen et al^24^ constructed a mechanical model with dual plasticity based on the consideration of solid coal plastic deformation and weakening of both sides' coal pillar width and support capacity in the basic roof structure. This model aimed to study the fracture mode and engineering guidance significance of the basic roof structure under the engineering conditions of leaving coal pillars in both sides' mined-out areas. Xie et al.^25^, Chao^26^ discovered that during the extraction stage of isolated working faces, a macroscopic stress shell forms in the surrounding rock mass of the goaf, serving as the main support system, bearing and transmitting the load of the overlying strata. Throughout the entire extraction process, the stress arches in the surrounding rock mass of the isolated mining face essentially undergo a dynamic evolution process of continuous “arch failure—arch stabilization”. Ming et al^27^, Huo et al^28^, and Feng et al^29^ used mechanical modeling and numerical simulation methods to propose the “arch shell” structure of the overlying rock in the mining area, and found that the stress arch experienced an evolution process of “arch formation arch height increase stage arch failure arch formation stability” during the mining stage.

Determination of brace working resistance is a key technology to ensure the safe mining of coal pillar in the section. Wang et al^30^ established a binary criterion for determining the working resistance of braces by balancing the roof load and keeping the coal wall stable. The dynamic load calculation method for calculating the determination of bracing working resistance for controlling the roof plate when the basic roof structure is unstable was proposed. Zhang et al^31^ called the movement of the direct top rock layer above the mining field as “necessary control rock layer”. The basic top rock layer is called “programme-controlled rock layer”, and derives the mathematical mechanical formula of the relationship between them and the rated working resistance of the stent, and then determines the working resistance of the stent.

The above research results are mainly for the conventional isolated island working face (working face width ≥ 150 m), the mining system of isolated island-type coal pillar is mostly for the specific engineering problems, stereotypical interpretation, due to the section of coal pillar by the adjacent working face of the superposition of the mining impact is more serious, the complexity of the conditions of endowment, the mining action of overburden spatial structure of rock interacting with each other, and the load-bearing structure is also more complex. At the same time, multiple disaster-causing effects are involved. In view of this, it is urgent to study the breakage law of overburden rock and safe mining technology to realize the safe and efficient recovery of the sectional coal pillar working face under the superimposed mining stress conditions. Based on the technical system of safe recovery of multiple types of legacy coal pillar resources in Zhaogu No.2 Mine, this paper comprehensively uses the research methods of field measurement, numerical simulation, theoretical analysis, engineering test and other research methods. It explores and researches the breaking law of overlying rock and safe opening technology of the sectional coal pillar working face, and provides theoretical guidance significance for the safe mining of the section coal pillar working face.

## Legacy coal pillar working face mining system

The diversity of coal mining conditions in China, as well as the limitations imposed by historical mining levels, have resulted in a large amount of residual coal resources^4,32–34^. The safe and efficient recovery of residual coal resources requires guidance from a comprehensive coal recovery technology system, closely related to the management and prevention of various disasters encountered during residual coal recovery. Due to the complexity of residual coal resource types and occurrence conditions, as well as the involvement of multiple disaster-causing effects, targeted mining theories and technologies are needed for residual coal under different occurrence conditions. So far, there is no mature theoretical and technical system in place.

**Figure 1:**
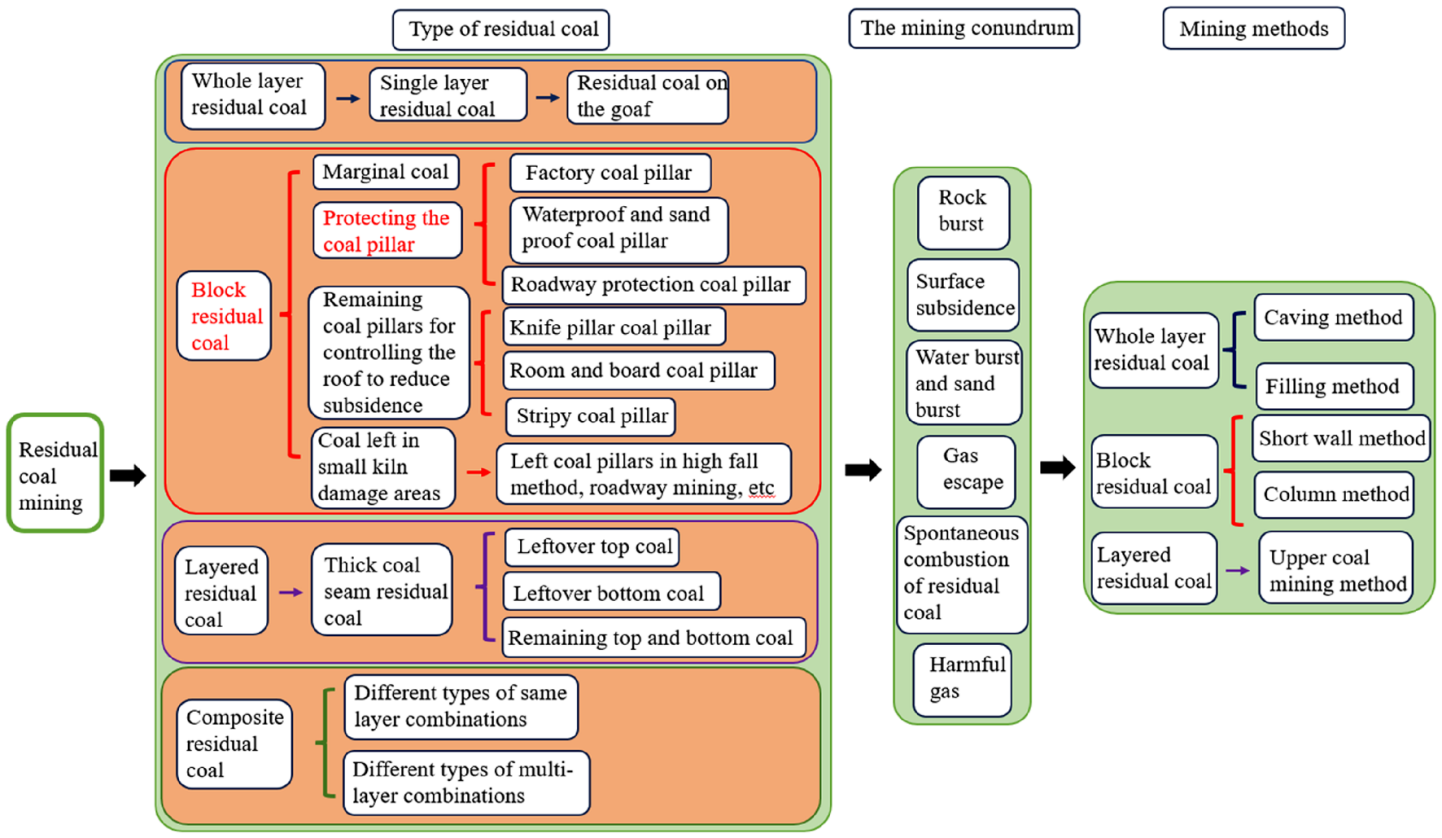
Legacy coal pillar mining framework.

In the field of shallow coal mining theory and technology in China, Feng et al.^4,35–37^ have conducted a significant amount of targeted research and achieved remarkable results. Feng et al^4^, through a nationwide survey of residual coal pillar resources, classified residual coal into three basic types: whole-layer residual coal, block-section residual coal, and stratified residual coal, as well as combinations of these three types, forming composite residual coal. They conducted research on different types of residual coal and proposed targeted mining methods and technologies, as shown in Fig. 1. They have made significant contributions to the exploitation of residual coal resources in China.

The segment coal pillar belongs to the block-section residual coal in residual coal, characterized by “short face length” and “concentration of stress due to superimposed mining”. Researching its mining methods, techniques, and the movement of overlying rock structures are key focuses in studying the recovery of this type of coal pillar.

**Figure 2:**
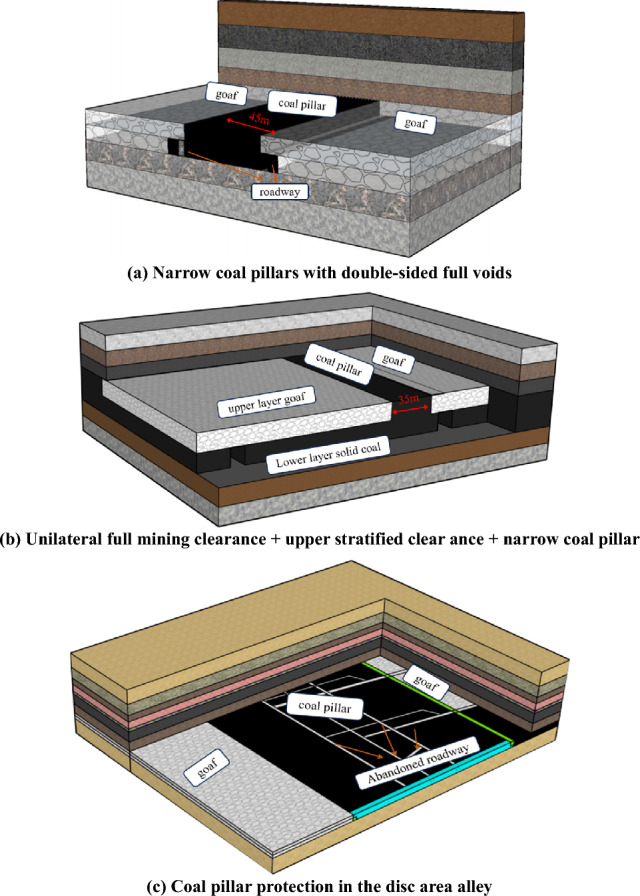
Types of residual coal pillars in mines.

Zhaogu No.2 Mine is limited to the mining technology and the conditions of the coal seam, which has produced a large number of high-quality thick coal seam residual coal pillars. The residual pillar resources in the mine primarily exist in three forms, as illustrated in Fig. 1: narrow coal pillars with full extraction on both sides (Fig. 2a), narrow coal pillars with partial extraction on one side and upper stratified extraction (Fig. 2b), and coal pillars protected in large roadway areas (Fig. 2c).

**Figure 3:**
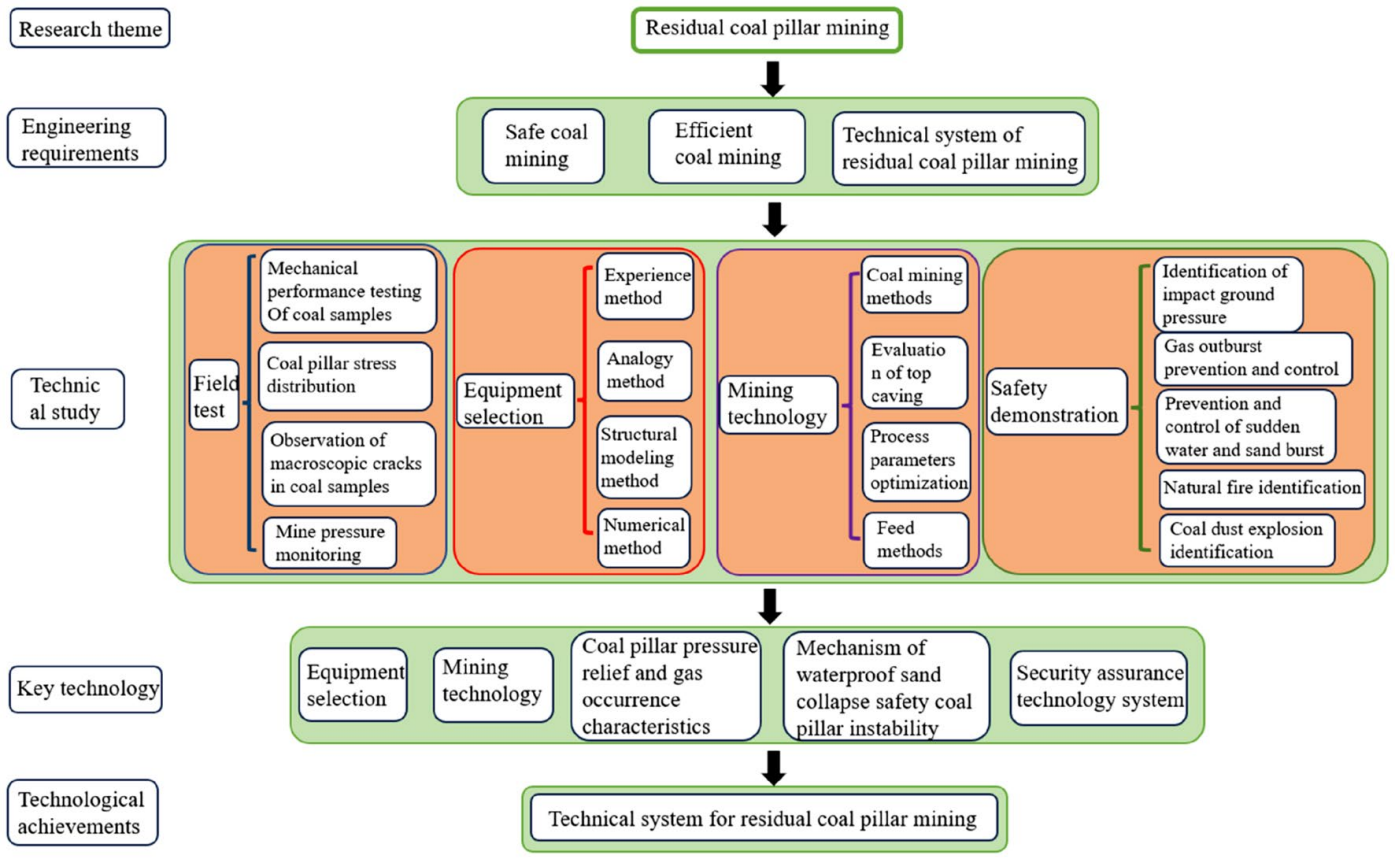
Technical framework for residual pillar mining.

Compared with the general working face, the residual mining face has the following characteristics: ① mining conditions are affected by the adjacent mining airspace, the working face stress concentration, with the risk of coal and gas protrusion, with the threat of impact pressure; ② the working face bedrock is thin, the mining of the threat of water and sand breakout; ③ surrounded by the airspace, harmful gases and water easily into the working face, the potential for secondary disasters; ④ The length of the working face is short and the conditions are complicated, so it is difficult to select the equipment and the mining process. The safe and efficient mining of residual coal pillars must solve the above problems. At present, systematic research has been carried out on three types of residual coal pillars in this mine, with good field application results, and all of them have been successfully mined back. Based on the conditions of the residual coal pillar, the requirements of safe and efficient coal production, through the testing of the mechanical properties of the pillar on site and data monitoring during the mining period, various methods of equipment selection, the study of the working face mining process and parameter optimization, and the safety demonstration of the residual coal pillar mining, etc., we have formed a set of residual mining technology system, which combines the selection of the equipment, the mining process, and the safety and technical guarantee as a whole, is shown in Fig. 3.

Based on the above residual mining pillar mining system, the dynamic evolution law of overburden rock structure of residual mining pillar is grasped to guide the safe and efficient mining of residual mining pillar under similar conditions. Limited to the length of the article, the author only focuses on the breakage law of the overburden rock structure of the narrow coal pillar with double-sided full-mining.

## Characteristics of damage to the working face of the coal pillar in the section

### Engineering background

**Figure 4:**
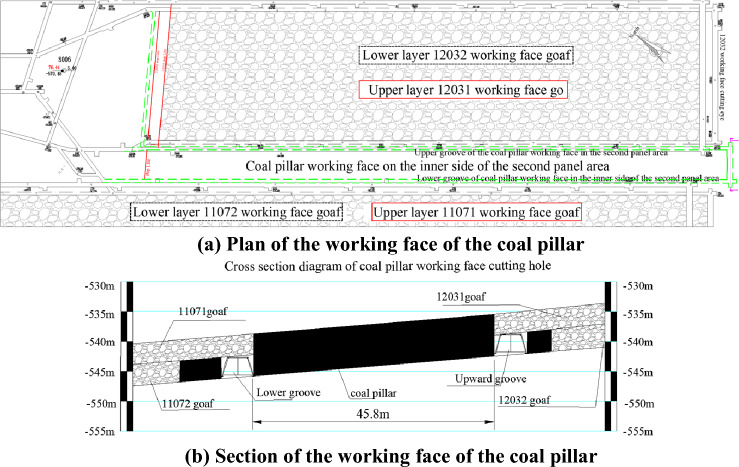
Coal pillar working face plan and section.

The coal pillar in the second disc area was originally a section of coal pillar to ensure the safe mining of the working face on both sides, and to support the overlying rock layer and maintain the stability of the roadway perimeter rock. The section coal pillar working face is located in the middle of the second panel area. The northeast side is the 12,031 and 12,302 working faces that have completed the mining, and the southwest side is the 11,071 and 11,072 working faces that have completed the mining. Both working faces adopt a layered mining method. To the northwest is the uphill return wind of the Erpan area, and to the southeast is the F_18_ fault water-resistant coal pillar,. The cross-sectional view of the working face is shown in Fig. 4.

The working face of the coal pillar on the inner side of the second panel is buried at a depth of 629.4–652 m, with an average minable strike length of 743.7 m and a width of 45.8 m. The main coal seam is the No. 21 coal seam, with a coal thickness of 5.8–6.9 m and an average coal thickness of 6.0 m. The coal seam has an inclination angle of 3–5°, and there are 1–2 layers of interbedded gangue within the coal seam, with a thickness of 0.03–0.5 m. The working face adopts a mechanized coal mining method with a backward strike long wall fully mechanized caving method, and the roof is managed using the natural collapse method.

**Figure 5:**
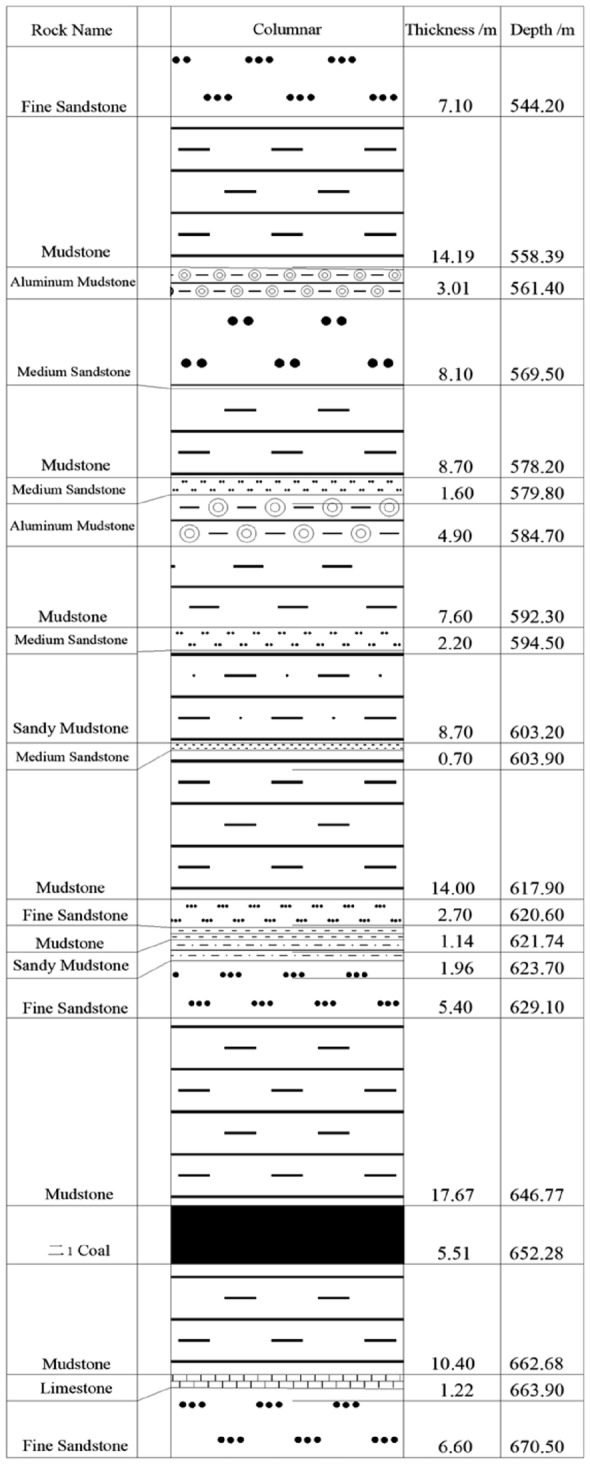
Column of the working face of the lining coal pillar in the second disc area.

The coal seam structure of the coal pillar working face is simple, the direct top of the working face is dominated by mudstone and fine-grained sandstone, and the old top consists of medium-grained sandstone with good stability, and the column shape of the coal pillar working face on the lining side of the second disc area is shown in Fig. 5.

As the working face is affected by the two sides of the mining airspace, the formation of stress concentration on the working face of the coal pillar, resulting in the working face back to the mining project, the coal body may be subject to a large static load or dynamic load, thus increasing the occurrence of the working face of the occurrence of impact ground pressure disaster.

### Analysis of pre-mining stress field in section coal pillar working face

Compared to conventional working faces, the manifestation of mining pressure in segment coal pillar working faces exhibits significant differences, mainly due to the different stress field distribution in segment coal pillar working faces. Therefore, understanding the initial stress distribution state before the extraction of coal pillar working faces is of great significance for studying issues such as stress transmission and overlying strata movement in coal pillar working faces.

**Figure 6:**
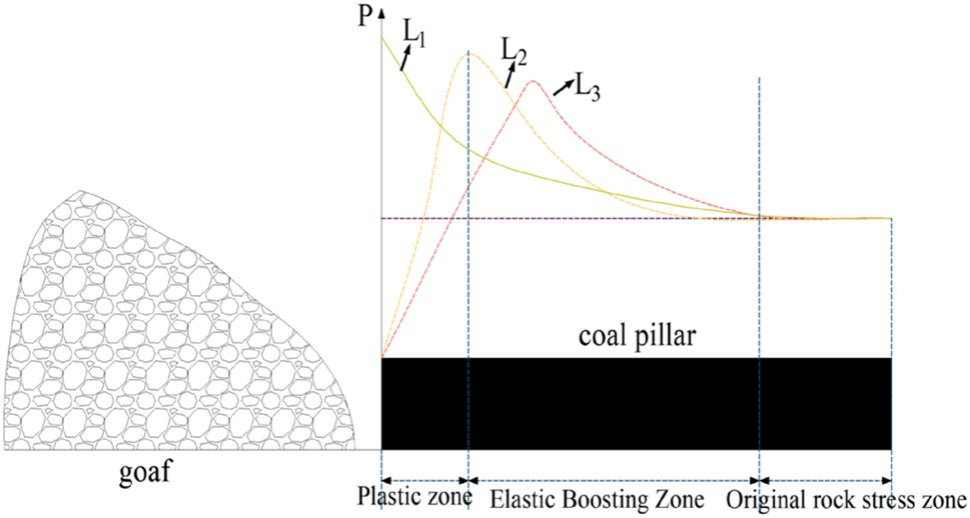
Distribution of lateral support stresses in coal pillars.

The concentration of stress in the coal pillar working face is mainly due to the influence of lateral support stress from the mined-out areas on both sides. The distribution of lateral support stress on one side of the coal pillar working face is illustrated in Fig. 6. During the early stages of coal pillar working face extraction, the lateral edge of the coal mass in the mined-out area reaches its strength limit. Due to low extraction intensity, the immediate roof has not yet fractured, and the coal mass remains undisturbed. The lateral support pressure exhibits a monotonically decreasing curve, as shown by L_1_ in Fig. 6.

As the working face advances, the overlying rock strata fracture and collapse, transferring the stored energy within the rock mass to the mined-out areas and solid coal pillars. Under the pressure of lateral support, the coal seam enters a plastic deformation stage, leading to a decrease in its bearing capacity. The lateral support stress transfers inward towards the coal pillar, as shown by L_2_ in Fig. 6. As the working face continues to advance, the immediate roof behind the mined-out area of the working face fractures, and the energy is once again transferred to the coal pillar, causing the lateral support pressure to transfer deeper into the coal mass, as illustrated by L_3_ in Fig. 6. Therefore, it can be determined that before the extraction of segment coal pillars, there is an accumulation of lateral support stress from the mined-out areas, resulting in stress concentration and deformation failure of the coal pillar, mainly within a certain range close to the adjacent roadways.

**Figure 7:**
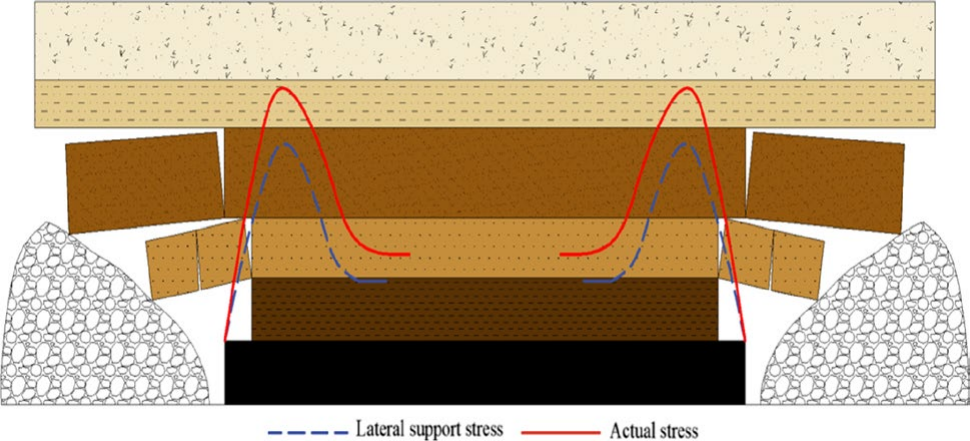
Stress distribution in the middle coal body after the two sides of the mine clearance.

After the extraction of the working face on the other side of the coal pillar, the stress curve of the coal pillar exhibits a similar pattern. When the two curves are superimposed, the coal pillar experiences the combined lateral support stress from both mined-out areas, resulting in significant stress concentration. The stress curve shows a bimodal pattern, indicating the stress distribution within the coal pillar between the two mined-out areas, as depicted in Fig. 7. Additionally, the lateral support stress curves from both sides of the working face overlap, but it is difficult to determine the formation of a stress accumulation zone in the middle of the coal pillar. Further numerical calculations and studies are required to ascertain this.

### Characteristics of fissure development within the coal pillar

**Figure 8:**
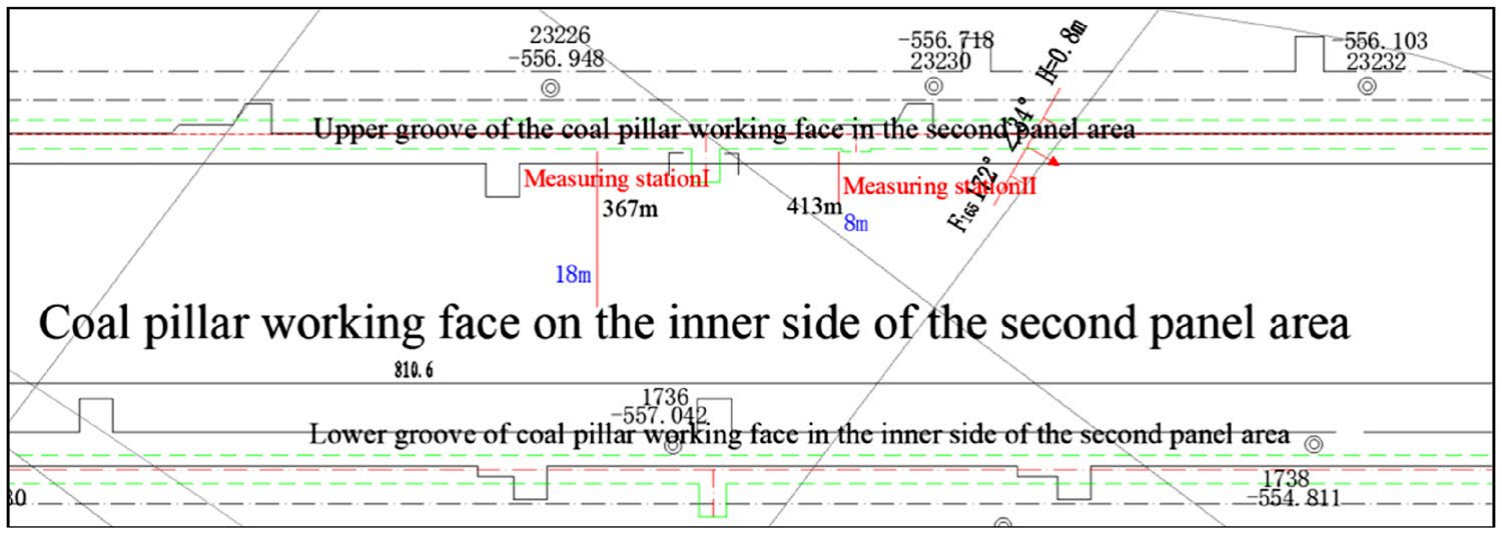
Observation Borehole Locations.

In order to observe the development of fissures in the coal body under the influence of superimposed mining on both sides of the working face, two peep holes were arranged in the coal pillar working face at the inner side of the second disc area to observe the development of fissures in the coal pillar working face. Observation holes 1 and 2 are located at 367 m and 413 m respectively from the starting position of roadway excavation, and the position of the drill holes relative to the stopping line of the working face is shown in Fig. 8.

**Figure 9:**
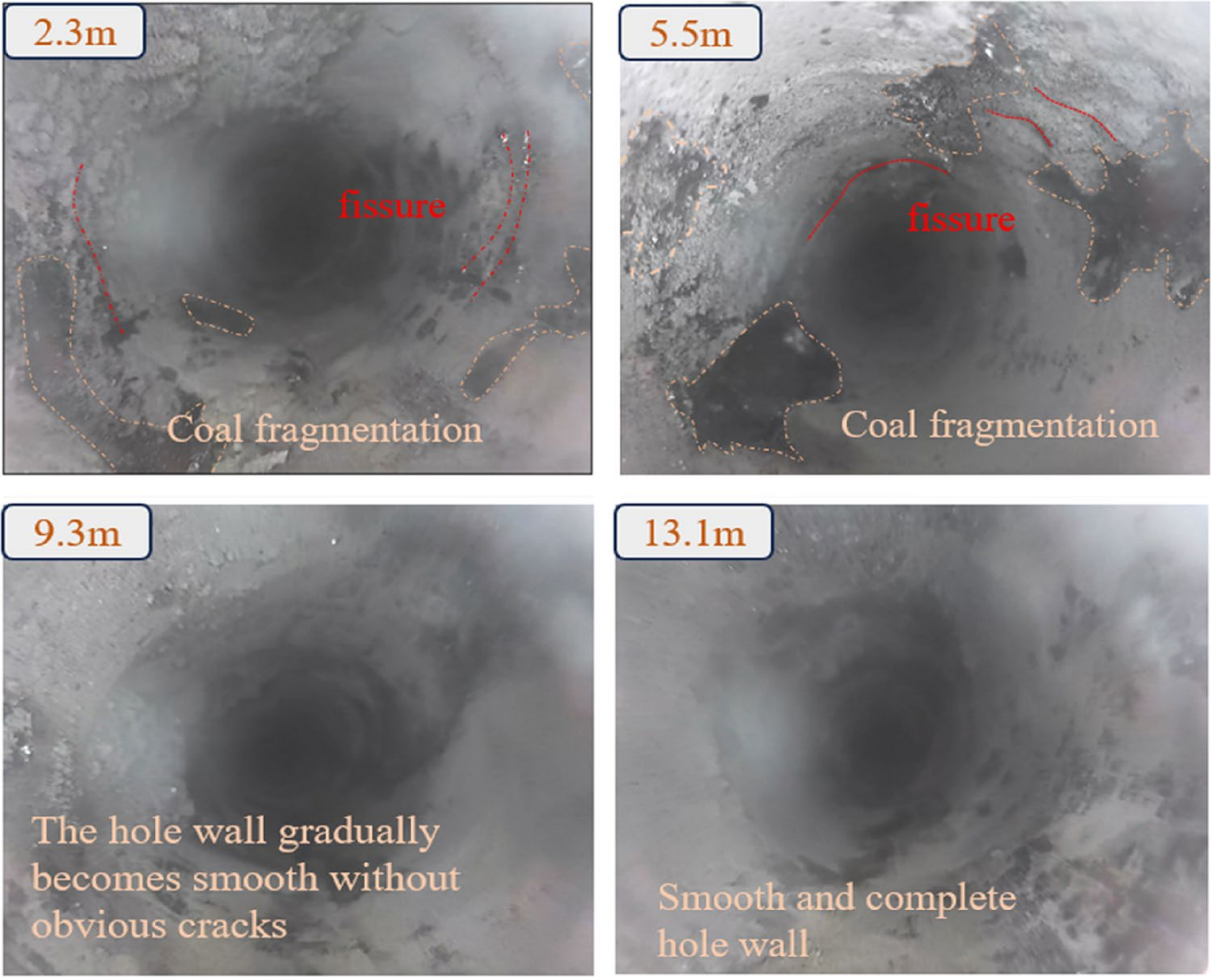
Drill hole peephole view of observation hole 1.

**Figure 10:**
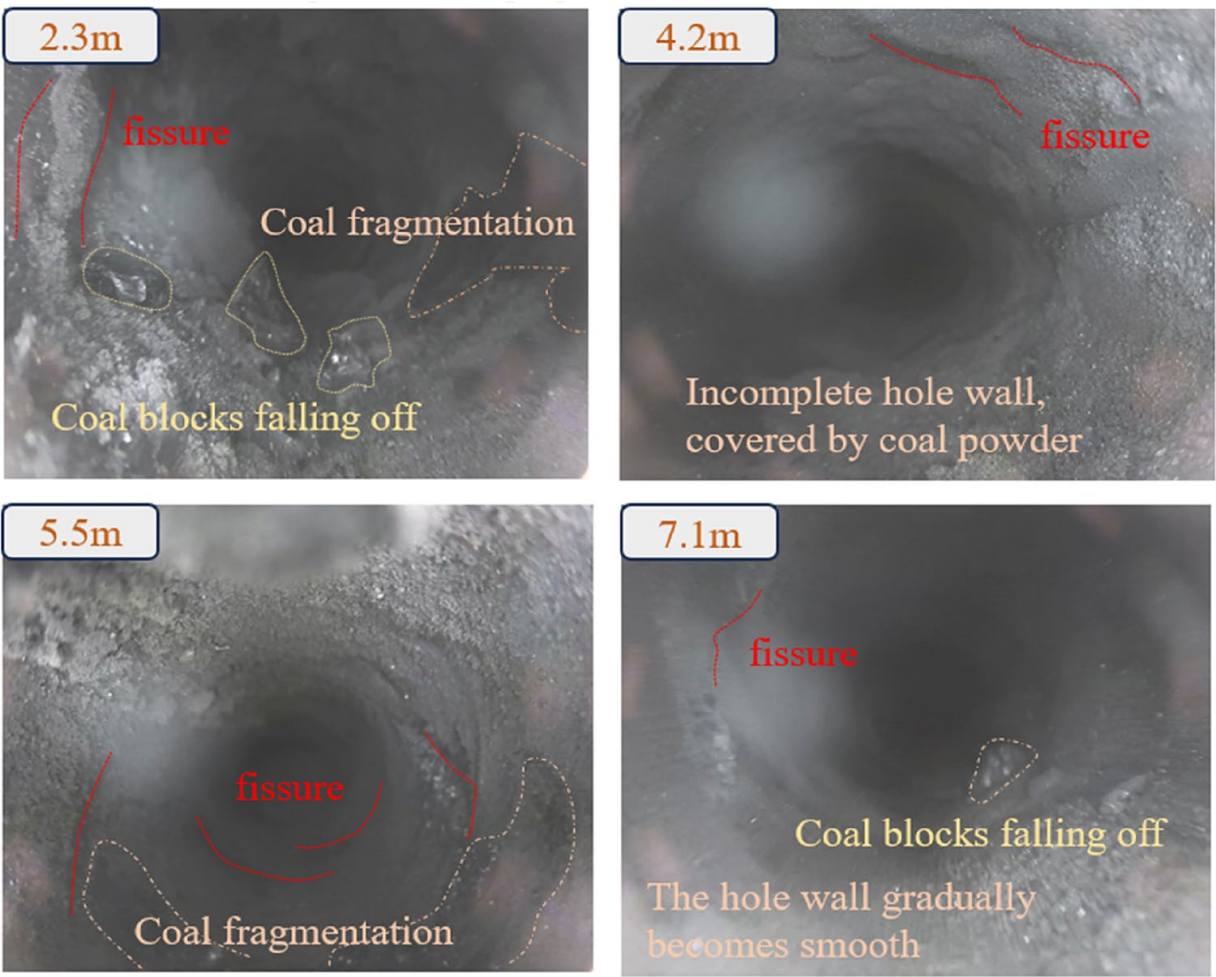
Drill hole peephole view of observation hole 2.

The drilling positions for the two boreholes are 1.5 m above the bottom of the groove, with a horizontal angle. The drilling depth is 8 m at a distance of 413 m from the starting point of the tunneling, and 18 m at the position 367 m away. During the construction process, collapses occurred multiple times. The development of fissures in the coal seams observed in Observation Hole 1 and Observation Hole 2 are illustrated in Figs. 9 and 10, respectively.

According to the image of the drill hole peeper, the wall of the drill hole up to 5.5 m range of fissure development, the wall of the drill hole broken, accompanied by the falling of coal pieces. With deeper peeping, the number of fissures in the borehole decreases, and the borehole wall is gradually complete. According to the result of peeping, it is known that a certain range of both sides of the coal body are affected by the disturbance of mining, which produces plastic damage, and the internal stress of the coal body is released, thus producing a large number of fissures. From this, it can be determined that the coal body of the working face of the section coal pillar is seriously damaged before mining, mainly in a certain range of the coal pillar from the two sides of the roadway closer to its internal fissure development, and the coal body shows the characteristics of fissure, separation, mining and so on.

## Spatial and temporal evolution law of overburden rock transport in coal pillar working face

### Numerical model

**Figure 11:**
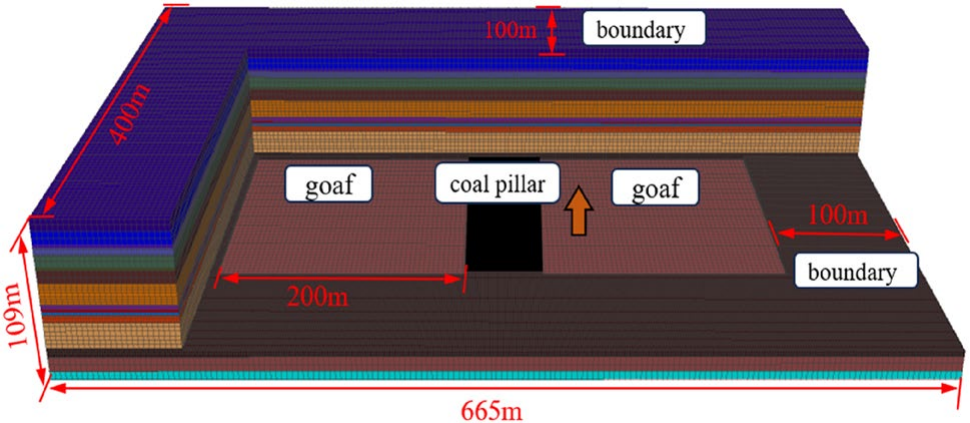
Three-dimensional numerical model diagram.

Based on the geological conditions of the working face in the inner coal pillar area, a three-dimensional model is constructed. To recreate the stress state of the working face of the sectioned coal pillar before extraction, working faces are established on both sides of the sectioned coal pillar. The length of each working face is 200 m, simulating an advancement distance of 200 m. The coal pillar is located in the middle with a width of 45 m. The model dimensions are 665 × 400 × 109 m (X × Y × Z), where the X-axis represents the direction of the working face, the Y-axis represents the direction of face advancement, and the Z-axis represents the strata. The overall three-dimensional model is illustrated in Fig. 11, and the model parameters are provided in Table 1. The average burial depth of the mining area is 650 m. For the upper strata, representing simulated rock layers, uniform loading is applied, while displacement is constrained at the boundaries and bottom. To mitigate boundary effects, boundary coal pillars of 100 m are established on all sides of the mining area. The constitutive relationship follows the Mohr–Coulomb criterion, and a double yield model is employed to simulate compaction effects in the goaf area. To enhance computational efficiency, excavation of working faces 11,071 and 11,072 will be conducted simultaneously, with a step of 10 m for each excavation, totaling 200 m. After stable extraction is achieved, working faces 12,031 and 12,032 will be excavated simultaneously. Finally, the working face of the sectioned coal pillar will be excavated.

**Table 1:** Numerical model parameter list.

Lithology	Poisson’s ratio	Elastic modulus/Gpa	Internal friction angle/°	Cohesion /Mpa	Tensile strength/Mpa
Medium sandstone	0.26	31	32.2	22.9	8.71
Sandy mudstone	0.30	28.5	32	18.5	6.2
Fine sandstone	0.25	50	37.8	34.5	8.79
Mudstone	0.33	16.3	32	15.4	5.34
Coal	0.32	23.1	34	14.8	2.6

### Analysis of simulation results before coal pillar workings are mined back

**Figure 12:**
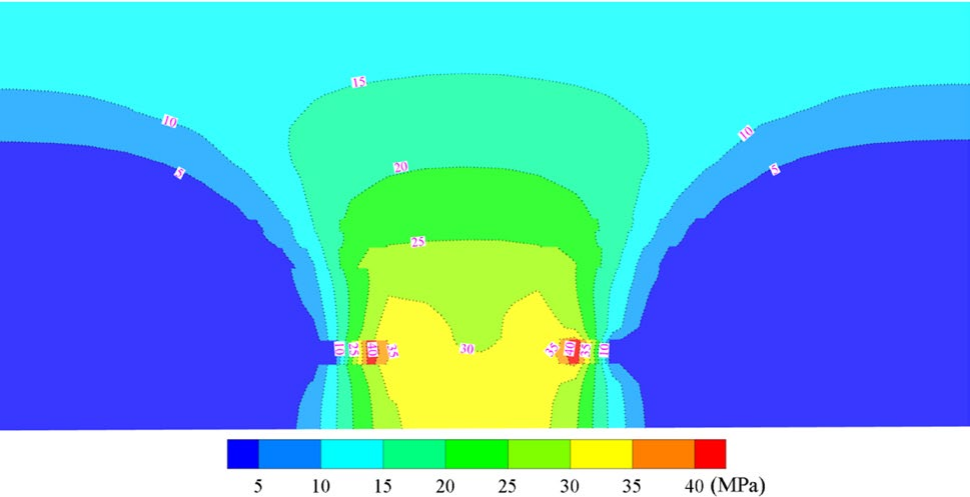
Vertical stress distribution cloud map of the middle coal pillar after the two sides of the mine clearance.

**Figure 13:**
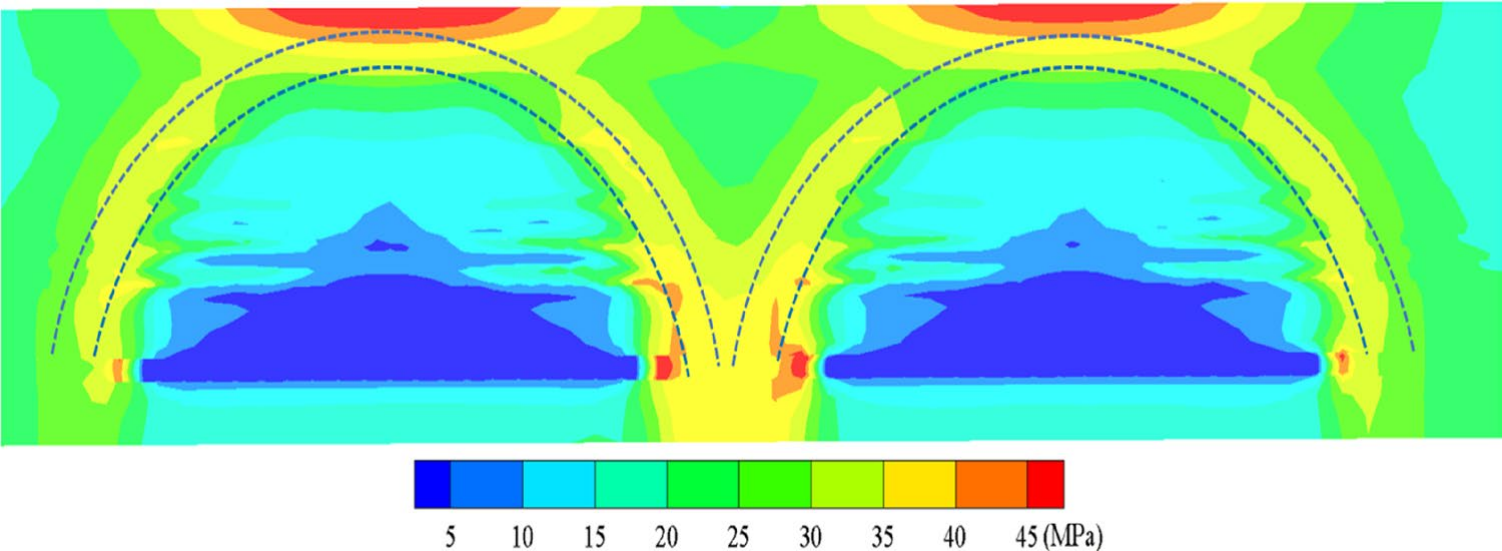
Cloud diagram of maximum principal stress distribution after two sides of extraction.

After the completion of mining in the two adjacent mining areas on either side of the sectioned coal pillar, the original rock stress in the three-dimensional model is disturbed, leading to a redistribution of stress. The vertical stress distribution in the coal pillar area after the two sides are mined out is illustrated in Fig. 12, while the distribution of maximum principal stress is depicted in Fig. 13. Stress arches form on both sides of the working faces, with the arch feet falling onto the coal pillar area. Consequently, before mining of the isolated island mining area, stress becomes highly concentrated. The stress is maximal on the two sides, relatively lower in the middle, exhibiting a bimodal distribution. The peak vertical stress is 43.7 MPa, with a trough value of 30.0 MPa, and the stress concentration factor ranges between 1.78 and 3.2.

### Analyses of simulation results under the state of mining back in the coal pillar working face

#### Mining stress distribution and evolution characteristics in the direction of the working face

**Figure 14:**
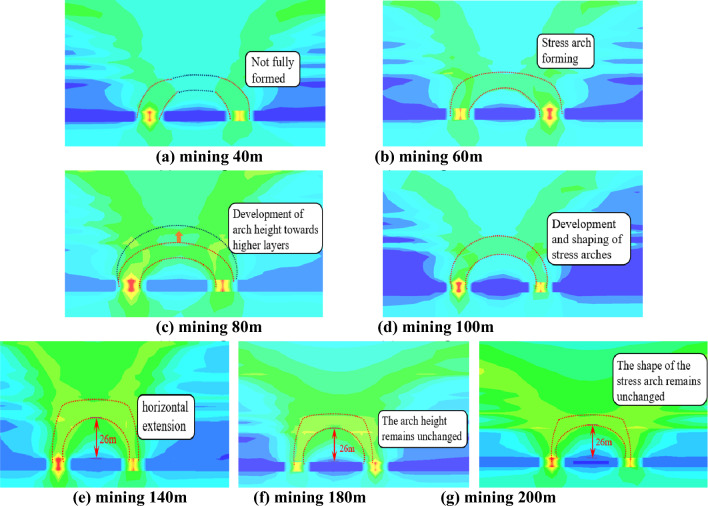
Dynamic evolution of the stress arch in the direction of the working face.

Statistical analysis of the distribution of maximum principal stress at 40 m, 60 m, 80 m, 100 m, 140 m, 180 m, and 200 m along the direction of the working face reveals the formation of stress arches composed of high stress zones on both sides and in the overlying strata, as depicted in Fig. 14. When the length of the working face advancement is 40 m, as shown in Fig. 14a, there is a noticeable area of unloading above the goaf, with significant stress concentration on both sides of the working face and relatively lower stress in the middle of the coal pillar. At this point, the stress arch has not fully formed yet. As the working face advances to 60 m, as illustrated in Fig. 14b, the stress arch develops fully. The formation of the stress arch represents the load-bearing effect of the overlying hard roof. Under the load-bearing action of the stress arch, the rock mass inside the arch fractures, and the working face mainly bears the load of the immediate roof, with minimal dynamic manifestation on the working face.

When the working face advances to 80 m, as shown in Fig. 14c, the stress concentration zone on the top of the arch moves upward. There are two stress concentration phenomena on the roof of the working face, and the stress values between the two arches are relatively low. At this point, the stress concentration zone on the top of the arch moves upward, and there is a tendency for the arch height to increase. As the working face advances from 80 to 100 m, reaching the limit span of the immediate roof, continuous fracturing and instability occur in the low-level immediate roof beams inside the arch, reducing their load-bearing capacity. The impact load on the working face support is further intensified, and the stress arch expands upward as the working face advances. This expansion continues until the stable evolution of the high-level roof beam structure, stabilizing the development of the stress arch. The impact of rock mass fracturing within the arch on the support is reduced, as illustrated in Fig. 14d.

After the formation of the upper stress arch, as depicted in Fig. 14d–g, with the advancement of the working face, the top of the stress arch only extends horizontally, and the shape of the stress arch remains essentially unchanged. There is still stress concentration at the top of the arch, and the maximum principal stress inside the arch remains relatively low. The height of the arch remains constant at 26 m.

**Figure 15:**
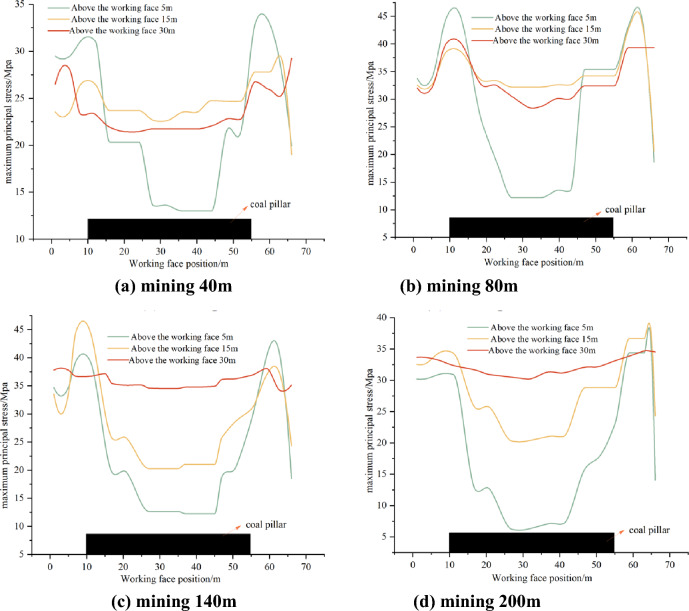
Maximum principal stress distribution pattern of the working face roof plat.

At positions 5 m, 15 m, and 30 m above the roof of the working face, measuring lines 1, 2, and 3 are respectively arranged. Statistical analysis is conducted on the maximum principal stress values at distances of 40 m, 80 m, 140 m, and 200 m along the advancement of the working face, as illustrated in Fig. 15. The working face advances to 40 m, as shown in Fig. 15a, the maximum concentration of stress is concentrated on both sides of the working face, i.e., the position of the arch foot, and the distance of its stress value decreases gradually with the rise of the working face top plate. The stress in the middle of the working face top plate is small compared with the two sides, with an average of 23Mpa, at this time, the stress in the middle of the working face is not yet concentrated. When the working face advances to 80 m, as shown in Fig. 14b, stress concentration occurs at the measuring line 2 and 3 above the working face roof, and the stress value is about 33Mpa, and “double arch” exists at the same time above the working face, which indicates that there is a tendency of upward transition of the stress arch to increase in height. As shown in Fig. 15c,d, the maximum principal stress value is larger only at the measurement line 3, and its stress value is basically unchanged, with a stress value of about 32Mpa, and the stress value at the measurement line 1 and 2 is small, which indicates that with the advancement of the working face, the location of the maximum principal stress concentration basically remains unchanged.

**Figure 16:**
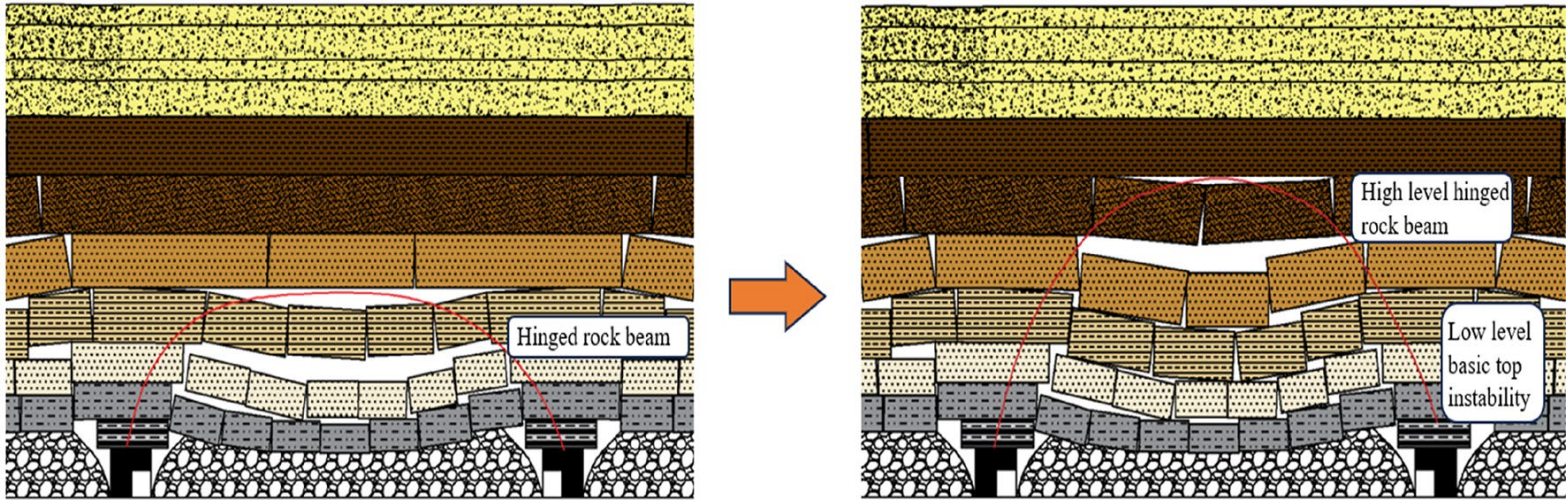
Evolution model of low to high arches in the working face.

In summary, the working face has experienced a low arch to high arch evolution process during the advancement of the working face, and the evolution process is shown in Fig. 16. In the process of mining back to the working face, the stress arch first experiences the evolution process of “development—formation—elevation”, at this time, the main low basic top plays a major role in bearing, with the advancement of the working face, the low basic top breaks off and destabilizes, and at the same time, the impact load is formed on the working face, and the stress arch expands upwards until the high articulated rock beam structure stabilizes, and the stress arch develops and forms to the high position. After that, the shape of stress arch is basically unchanged. Generally speaking, the stress arch of the coal pillar face undergoes the evolution process of “development—formation—elevation—stability”.

#### Workface strike mining stress distribution and evolution characteristics

**Figure 17:**
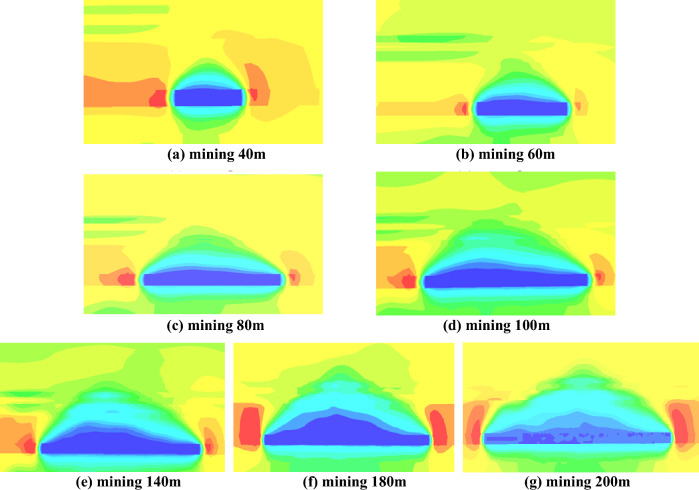
Dynamic evolution of the stress arch in the advancing direction of the working face.

Figure 17 shows the evolution cloud map of the maximum principal stress field of the surrounding rock in the middle of the working face along the direction of the working face. From the figure, it can be seen that there are stress arches in the surrounding rock along the direction of the working face. The arch foot is located in front and behind the working face. Due to the influence of the combined mining on both sides and the advance support stress of the working face, a large range of high-concentration stress appears in front of the coal wall, which is significantly different from non-island working faces. When the advancing distance of the working face is less than 80 m, the stress arch develops to a height of 17 m, extending to the basic roof, with the main load of the working face borne by the basic roof. Once the working face advances beyond 80 m, the lower basic roof fractures and collapses, causing the stress arch to develop upwards, extending to the hard rock layer at the higher level. As the working face continues to advance, the range and intensity of maximum principal stress concentration in the surrounding rock of the mining field tend to stabilize, with the shape of the stress arch remaining essentially unchanged.

**Figure 18:**
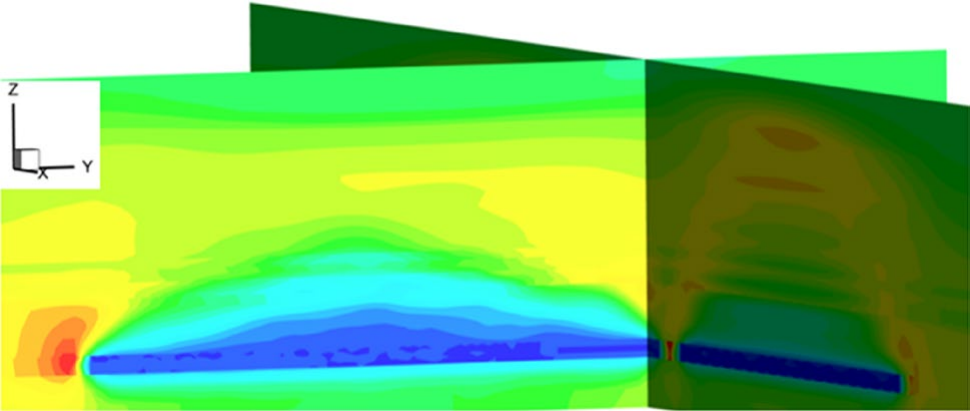
Distribution pattern of the maximum principal stress field in the quarry perimeter rock.

As shown in Fig. 18, the distribution pattern of the maximum principal stress field in the surrounding rock of the mining field indicates that stress arches appear both in the strike and dip directions of the working face. Throughout the entire mining field, there is a stress shell resembling an ellipsoid. The stress shell is the primary load-bearing structure of the surrounding rock, with the working face located in a low-stress area beneath the stress shell. Controlled by the protective effect of the stress shell, dynamic pressure during the mining process at the working face is not significantly evident.

## Structural study of overburden stress arch in coal pillar working face

**Figure 19:**
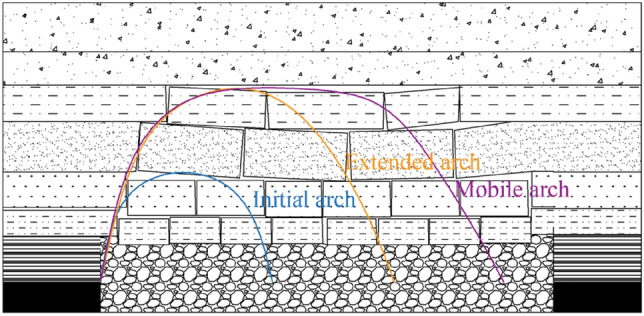
Schematic diagram of the structure of the overburden bearing arch of the quarry.

According to the numerical simulation results, stress arches appear in both the strike and dip directions of the working face within the entire mining field, forming a stress shell resembling an ellipsoid. As the working face advances, the lower basic roof destabilizes and fractures, causing the stress arch to expand upwards until the higher hinged rock beam structure stabilizes. The stress arch then develops upwards, as depicted in Fig. 19.

**Figure 20:**
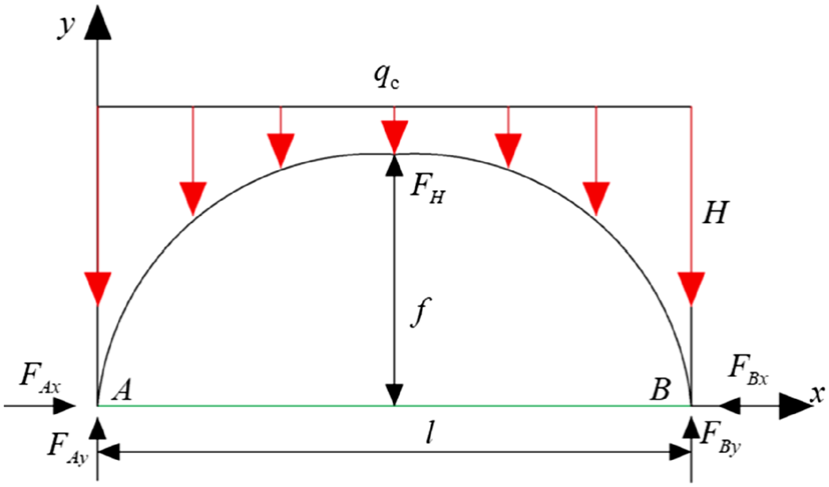
Mechanical model of a three-hinged arch.

The stress arch of the working face overlying strata is simplified into a three-hinged arch structure, and a stress arch mechanical model is established as shown in Fig. 20.

where *F*_*Ax*_, *F*_*Bx*_ represent the horizontal stress at the arch foot, in MN; *F*_*Ay*_, *F*_*By*_ represent the vertical stress at the arch foot; *F*_*H*_ represents the horizontal stress at the arch crown, in MN; *q*_*c*_ represents the concentrated load on the overlying strata, in MN; the height of the arch is denoted by *f*, and the span by *l*.

1 $$ F_{H} = \tan \varphi F_{Ay} $$

2 $$ F_{Ay} = \frac{{2q_{c} + \gamma y}}{2} $$

where *φ* represents the internal friction angle of the overlying rock layer, and tan*φ* represents the friction coefficient.

Let *M*^*0*^ be the moment at any point of a simply supported beam under the same load conditions. Therefore, the expression for the moment at any point on the arch is:

3 $$ M = M^{0} - F_{H} Y $$

That is:

4 $$ Y = \frac{{M^{0} }}{{F_{H} }} $$

Differentiating both sides simultaneously yields:

5 $$ \frac{{d^{2} y}}{{dx^{2} }} = \frac{1}{{F_{H} }} \times \frac{{d^{2} M^{0} }}{{dx^{2} }} $$

Taking *q(x)* as the load intensity per unit length along the horizontal line, then:

6 $$ \frac{{d^{2} M^{0} }}{{dx^{2} }} = q(x) $$

7 $$ \frac{{d^{2} y}}{{dx^{2} }} = \frac{q(x)}{{F_{H} }} $$

Substituting $q(x) = q_{c} + \gamma y$ into Eqs. (6) and (7) yields:

8 $$ \frac{{d^{2} y}}{{dx^{2} }} - \frac{\gamma }{{F_{H} }}y = \frac{{q_{c} }}{{F_{H} }} $$

Solve this differential equation:

9 $$ y = Ach\sqrt {\frac{\gamma }{{F_{H} }}} x + Bsh\sqrt {\frac{\gamma }{{F_{H} }}} x - H $$

Substituting the boundary condition: when *x* = 0, *y* = 0 Therefore:

10 $$ A = \frac{{q_{c} }}{\gamma } $$

When *x* = 0, the shear force is 0, at which time *B* = 0. Therefore, the equation of the arch axis can be obtained from any point (*x*, *y*) on the arch:

11 $$ y = H(ch\sqrt {\frac{\gamma }{{F_{H} }}} x - 1) $$

where: *γ* is the original rock stress; *A*、*B* are parameters to be determined; ch is the hyperbolic cosine function $\cosh x = \frac{{e^{x} + e^{ - x} }}{2}$。

Associatively (1), (2), (11), the stress arch arch height can be expressed as:

12 $$ f = H\left( {ch\sqrt {\frac{\gamma x}{{(2q_{c} + \gamma y)\tan \varphi }}} - 1} \right) $$

**Figure 21:**
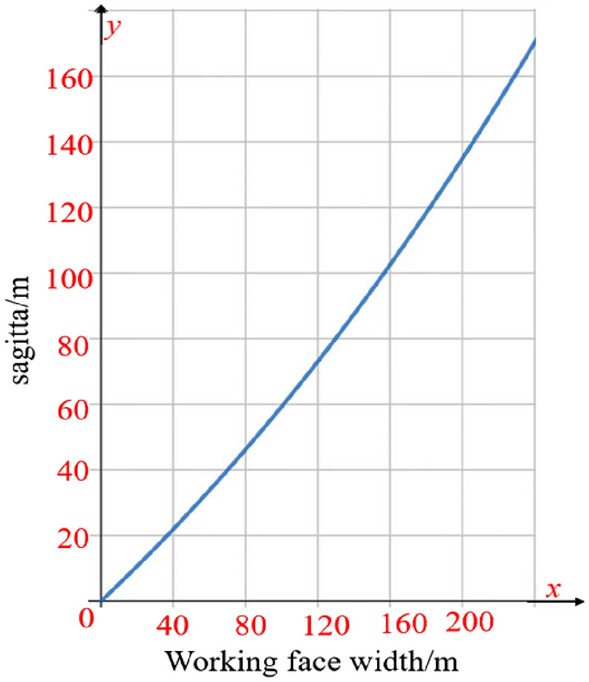
Relationship between working face width and arch height.

**Figure 22:**
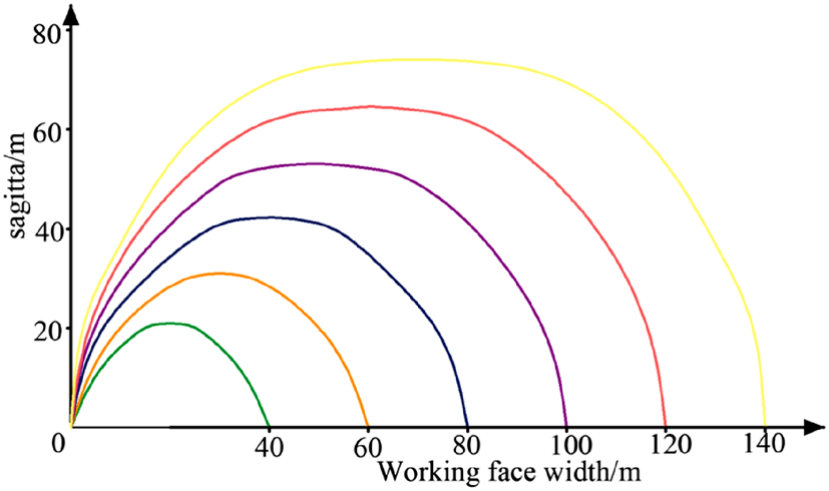
Overburden stress arch morphology.

According to the arch axis equation and the expression for the maximum arch height, the relationship between the width of the working face and the maximum arch height can be determined. The relationship diagram is shown in Fig. 21, while the stress arch shape of the overlying strata is depicted in Fig. 22.

As can be seen from the figure, the width of the working face has a great influence on the morphology of the stress arch, as the width of the working face increases, the larger the span of the corresponding arch in the bedrock section, the easier it is for the top rock layer to break and collapse, and the smaller the load of the overlying rock layer that the corresponding arch has to bear.

**Figure 23:**
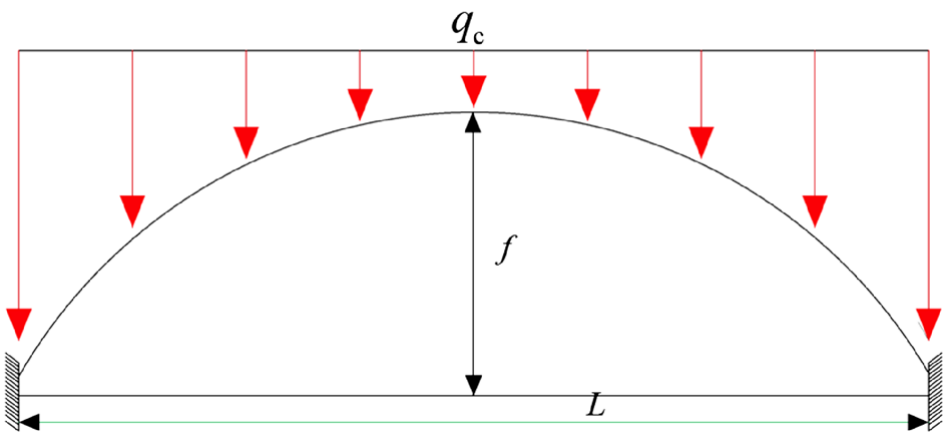
Mechanical model of the stress arch in the thrust direction.

On the working face strike, its stress boundary is different from that on the tendency, the location of the arch foot is in the position before and after the hollow area, and the two ends of the arch foot are solidly supported, and the mechanical model of the working face strike stress arch is established as shown in Fig. 23.

**Figure 24:**
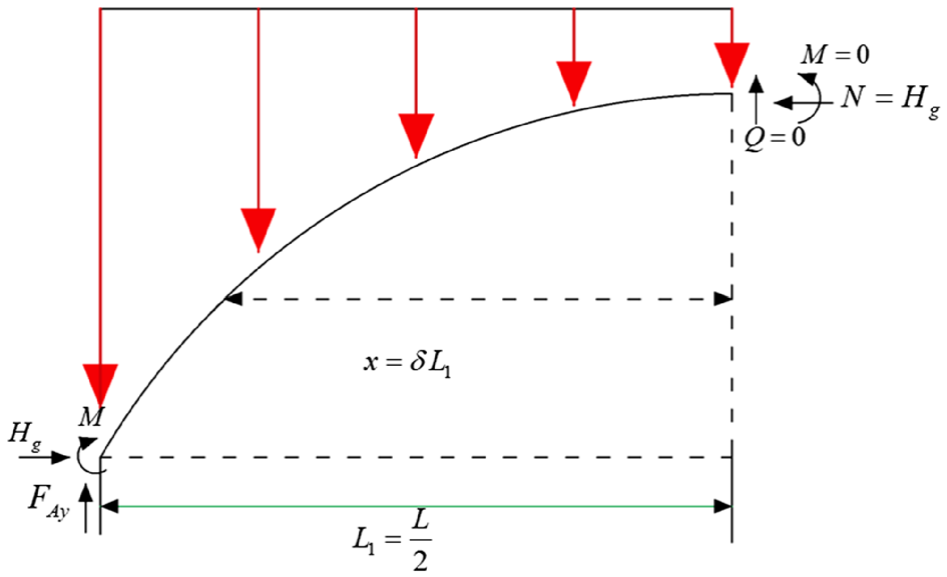
Sketch of stress analysis of the left half of the stress arch.

The arch axis is taken as the constant load pressure line, due to the principle of symmetry, positively symmetrical structure under symmetrical loading, the moment shear at the symmetry axis is 0. Since the pressure-bearing arch is a symmetrical model, one side is taken for the analysis, as shown in Fig. 24, the upper part of the arch is uniformly loaded with q, and the horizontal tangential support at the top of the arch is N.

The moment taken for the arch foot section is:

13 $$ H_{g} = \frac{M}{f} $$

The torque taken for any section is:

14 $$ y_{{}} = \frac{Mx}{{H_{g} }} $$

Taking two derivatives of x simultaneously for both sides of Eq. (11):

15 $$ \frac{{d^{2} y_{{}} }}{{dx^{2} }} = \frac{1}{{H_{2} }} \times \frac{{d^{2} M}}{{dx^{2} }} = \frac{{g_{x} }}{{H_{g} }} $$

**Figure 25:**
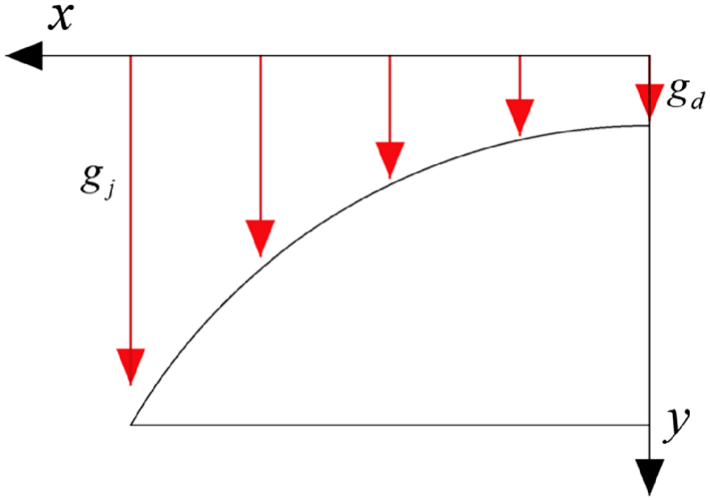
Non-linear load distribution.

Setting position parameters $\delta = \frac{x}{{l_{1} }}$ 。where *g*_*x*_ is the load at any point on the arch, the schematic diagram of the calculation model is shown in Fig. 25.

Calculation of the load on the arch:

16 $$ g_{x} = g_{d} + \gamma \cdot y $$

17 $$ g_{j} = g_{d} + \gamma f = mg_{d} $$

18 $$ \gamma = (m - 1)g_{d} /f $$

where *g*_*d*_ is the minimum load on the arch;* g*_*j*_ is the maximum load on the arch; *m* is the arch axis coefficient *m* = *g*_*j*_*/g*_*d*_.

Joining Eqs. (15), (16), (17), and (18), the load at any point in the x-direction can be obtained as:

19 $$ g_{x} = g_{d} + \gamma_{1} = g_{d} \left[ {1 + \left( {m - 1} \right)\frac{y}{f}} \right] $$

20 $$ \frac{{d^{2} y_{{}} }}{{ds^{2} }} = \frac{{l_{1}^{2} g_{d} }}{{H_{g} }}\left[ {1 + \left( {m - 1} \right)\frac{{y_{{}} }}{f}} \right] $$

Let:

21 $$ k^{2} = \frac{{l_{1}^{2} g_{d} }}{{H_{g} f}}(m - 1) $$

Substituting Eqs. (20) into (19) yields:

22 $$ \frac{{d^{2} (y)}}{{ds^{2} }} = \frac{{l_{1}^{2} g_{d} }}{{H_{g} }} + k^{2} y_{{}} $$

Solve this second-order non-homogeneous differential equation to obtain the equation of the axis of the arch in the direction of the working surface.

23 $$ y_{{}} = \frac{f}{m - 1}(chk\delta - 1) $$

where chkδ is the hyperbolic cosine function $chk\delta = \frac{{e^{k\delta } + e^{ - k\delta } }}{2}$.

According to the stress state and parameters equation of the pressured arch, the mechanical state at any position on the trajectory of the pressured arch can be obtained. At the beginning of mining, after the foot of the arch is located behind the open cut eye on the solid coal, the stress structure of the pressured arch undergoes initial instability. Subsequently, the foot of the arch moves forward to the compacted gangue in the goaf after the initial instability of the pressured arch. As the working face continues to evolve and develop in the surrounding rock, it affects the mining area. The pressured arch expands and forms a larger span and greater height pressured arch structure in the overlying strata at different stages of evolution.

## Determination of working resistance of working face support

Hydraulic supports play a crucial role in the movement and control of the roof strata in the working face, especially for isolated coal pillar working faces. The rational selection of hydraulic supports is a key factor in determining whether the working face can achieve safe and efficient production. Based on the characteristics of the stress arch structure, the support strength of hydraulic supports and the working resistance are calculated for the working face. Simultaneously, the adaptability of hydraulic supports is analyzed based on measured data during the mining period of the working face, providing reference for mine pressure control.

### Calculation of the strength of hydraulic support

**Figure 26:**
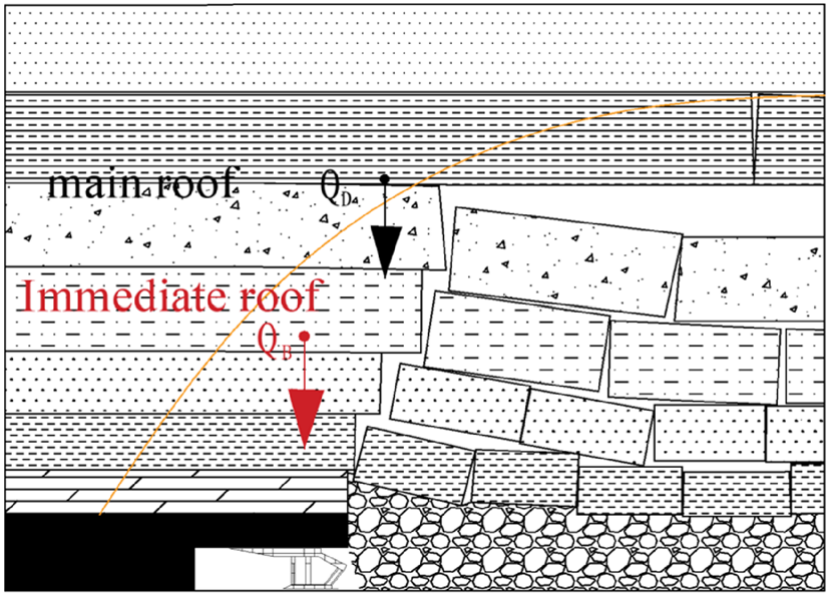
Calculation model of working resistance of bracket.

Due to the special storage conditions of the isolated coal pillar working face, the stress concentration of the coal pillar is obvious before mining back, but in the process of mining back, due to the bearing role of the stress arch, the load of the overlying rock layer is transferred to the two sides of the working face, forming the front and rear arches in the stress concentration area of the coal seam and the mining airspace area, and the working face is always located in the stress reduction area under the stress arch in the process of mining. Therefore, although the coal pillar is located in the stress concentration area, due to the bearing effect of the stress arch, the working resistance supported by the support is much smaller than the overall weight of the cover. The load of the working face support mainly comes from the self-weight of the rock layer in the stress reduction area and the dynamic impact of the basic roof instability on the support. The calculation model of brace working resistance is shown in Fig. 26.

Therefore, the working resistance of the support P is the sum of the gravity force of the direct roof within the range of the roof control distance and the impact load force on the support when the basic roof rock mass slides down and becomes unstable. The calculation formula is as follows:

24 $$ p = Q_{D} + K_{d} {\cdot}Q_{B} $$

25 $$ Q_{B} = H \times B \times \gamma \times L_{B} \times \cos \beta $$

26 $$ Q_{D} = h \times B \times \gamma \times L \times \cos \beta $$

where *Q*_*B*_—is the direct top gravity, kN; *K*_*d*_—is the dynamic load factor, take 1.2; *Q*_*D*_—is the basic top gravity, kN; *h*—is the thickness of direct top, m; *B*—is the width of the centre distance of the support, 1.5 m; *γ*—is the overlying rock layer capacity, 26 kN/m^3^; *L*— is the distance between the top of the stent and the centre of the stent, 4.1 m; *β*—is the inclination angle of coal seam, 5°; *H*—is the thickness of basic top rock layer and load layer, m.

Based on the relationship between stress arch and working face width, it is determined that the coal pillar's stress arch development height is 23.2 m. Combined with borehole columnar data, it is known that the stress arch develops to fine-grained sandstone at 2.74 m, and the lower basic roof is fine-grained sandstone at 5.4 m. Therefore, the load borne by the support is the sum of the self-weight of the strata in the stress reduction zone and the impact from the controlled load-bearing layer of the basic roof.

Calculated by substituting the data into Eqs. (24), (25) and (26):

$$ \begin{gathered} p = Q_{A} + K_{d} {\cdot}Q_{B} = 17.67 \times 1.5 \times 26 \times 4.1 \times \cos 5^{o} { + } \hfill \\ { 1}{\text{.2}} \times 8.5 \times 1.5 \times 26 \times 16 \times \cos 5^{o} \hfill \\ \, = 9153.48{\text{kN}} \hfill \\ \end{gathered} $$

Based on the above, it is calculated that the working resistance of the support should not be less than 9153.48 kN. Considering the actual situation of the supports in Zhao Gu Er Mine, the final decision is to use ZF10000/22/35D hydraulic supports for the inner coal pillar of the Second Panel area.

### Field monitoring and data analysis

**Figure 27:**
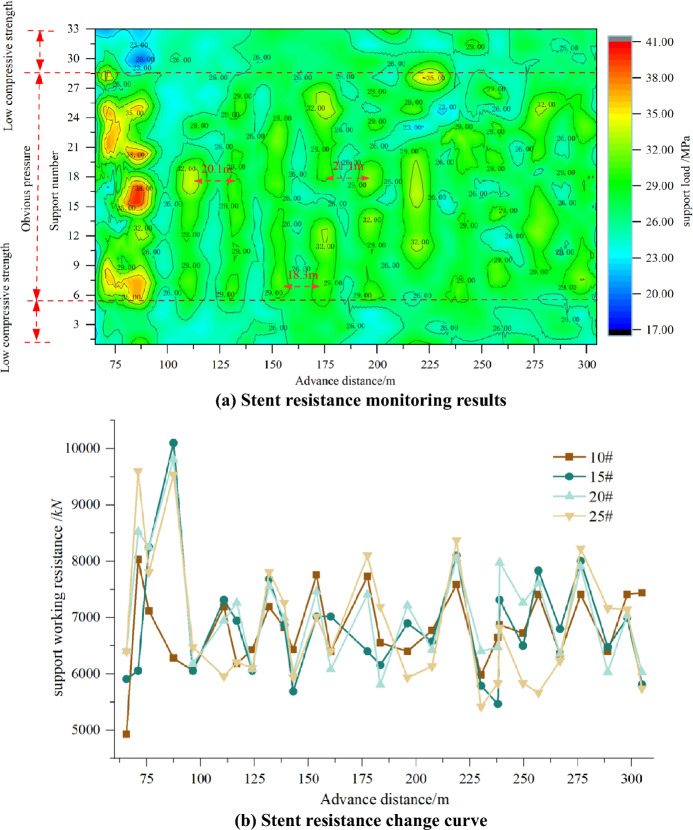
Pressure distribution of working face support.

The electro-hydraulic control system of the working face stent can display the stent pressure data in real time, and monitor and record the stent stress through the stress gauge. In the normal mining stage, take the working face from 65.6 to 304.8 m as an example to analyse the law of mine pressure, the working face stent pressure distribution is shown in Fig. 27, from the monitoring data in Fig. 27a, it can be seen that during the period of mining in the working face, the pressure area is obvious, the pressure in the middle of the working face is obvious, the pressure at the both ends is calm, and the pressure is obvious when the working face is pushed forward to twice the width of the working face, and the max. pressure can reach 41 MPa, the stent working resistance is 10,098 kN, after the working face is advanced to twice the width of the working face. The working resistance of the support is 10,098 kN, after the working face advances to twice the width of the working face, the coming pressure is not strong, within the rated working resistance of the liquid support. When the working face advances to two times the width of the working face, the top of the basic roof above breaks to form an impact on the working face, resulting in the working face of the mine pressure is obvious. Subsequently, the load on the working face is mainly carried by high-level articulated rock beams, leading to a decrease in pressure intensity on the working face. This indicates that the existing ZF10000/22/35D supports can meet the roof control requirements of the working face, effectively ensuring the safe production of the working face.

## Conclusions

- (1) Before the extraction of the sectional coal pillar, the coal body exhibits characteristics such as fragmentation and separation. Above the coal pillar, a “double-peak” stress distribution pattern is observed, with the maximum vertical stress reaching 43.7 MPa, and the stress concentration coefficient ranging from 1.78 to 3.2.
- (2) During the extraction of the sectional coal pillar, there is a stress arch composed of high-stress bundles in both the trend and direction of the overlying strata. This stress arch support structure undergoes a dynamic evolution process of “formation—development—rise—stability” as the working face advances. After the instability and fracture of the low-level basic top articulated structure, the stress-bearing structure shifts to the high-level basic top, serving as the primary support structure for the mining space of the working face.
- (3) According to the stress-bearing structure of the surrounding rock in the working face, a mechanical model of the stress arch in the quarry is constructed, and the equations of its inclination and direction to the arch axis in the working face are deduced, so as to grasp the morphological characteristics of the stress arch of the overlying rock in the working face of the coal pillar.
- (4) Combined with the coal pillar working face peripheral rock stress distribution law and stress arch evolution characteristics, through theoretical calculations, the maximum working resistance of the working face bracket is 9153.48 kN, and compared and analyzed with the measured mine pressure data, the selected bracket effectively guarantees the safe production of the working face.

## Data Availability

Some or all data, models, or codes generated or used during the study are available from the corresponding authors by request.
